# Optimising Treatment Delivery and Reducing Length of Stay in an Adult In-Patient Unit

**DOI:** 10.1192/bjo.2022.416

**Published:** 2022-06-20

**Authors:** Urvashnee Singh, Fiona Salter, Fiona Cartwright

**Affiliations:** Ramsay Clinic Hollywood, Perth, Australia

## Abstract

**Aims:**

Lifetime prevalence of eating disorders worldwide is 8.4% (3.3–18.6%) for women and 2.2% (0.8–6.5%) for men and this prevalence has been increasing over time. Anorexia nervosa has become a greater burden on secondary care: Not only have admission rates increased, but so too have multiple admissions per person with Anorexia Nervosa. Conservative treatment approaches and long lengths of stay have both direct and indirect costs for patients, hinder access to the service for potential patients and reduce service cost effectiveness. Ramsay Clinic Hollywood is a voluntary, private ten bed adult eating disorder inpatient service in Perth, Western Australia (WA). It is the only inpatient eating disorder specialist service for people over the age of 16, in both the private and public sector in WA. Over the past eight years, our focus has been on optimizing treatment delivery to minimise time spent in hospital for individuals with anorexia. The aim of this study was to evaluate whether instituting a rapid refeeding protocol was effective in optimising treatment outcomes, such as rate of refeeding and reducing length of stay (LOS).

**Methods:**

A retrospective review of data collected for all inpatients from 2013–2019 was conducted. The outcomes evaluated were length of stay and number of readmissions.

**Results:**

Utilising a rapid refeeding protocol successfully increased the rate of refeeding from 0.6kg/week to 1.5kg/week. This led to a reduction in average length of stay from 52 days in 2013 to 24 days in 2017. Concomitantly we have been able to double the number of patients admitted to the service/ year and reduce the number of readmissions/ patients

**Conclusion:**

These results suggest that it is possible to lower length of stay by increasing the rate of refeeding and this in turn allows more patients access to hospital care for their eating disorder.

